# Effect of selective serotonin reuptake inhibitor treatment following diagnosis of depression on suicidal behaviour risk: a target trial emulation

**DOI:** 10.1038/s41386-023-01676-3

**Published:** 2023-07-28

**Authors:** Tyra Lagerberg, Anthony A. Matthews, Nanbo Zhu, Seena Fazel, Juan-Jesus Carrero, Zheng Chang

**Affiliations:** 1 Department of Medical Epidemiology and Biostatistics, Karolinska Institutet, Stockholm, Sweden; 2 Department of Psychiatry, Warneford Hospital, University of Oxford, Oxford, UK; 3 Unit of Epidemiology, Institute of Environmental Medicine, Karolinska Institutet, Stockholm, Sweden; 4 Oxford Health NHS Foundation Trust, Oxford, UK

**Keywords:** Outcomes research, Depression

## Abstract

There is concern regarding the impact of selective serotonin reuptake inhibitors (SSRIs) on suicidal behaviour. Using the target trial framework, we investigated the effect on suicidal behaviour of SSRI treatment following a depression diagnosis. We identified 162,267 individuals receiving a depression diagnosis aged 6–59 years during 2006–2018 in Stockholm County, Sweden, after at least 1 year without antidepressant dispensation. Individuals who initiated an SSRI within 28 days of the diagnosis were assigned as SSRI initiators, others as non-initiators. Intention-to-treat and per-protocol effects were estimated; for the latter, individuals were censored when they ceased adhering to their assigned treatment strategy. We applied inverse probability weighting (IPW) to account for baseline confounding in the intention-to-treat analysis, and additionally for treatment non-adherence and time-varying confounding in the per-protocol analysis. The suicidal behaviour risk difference (RD), and risk ratio (RR) between SSRI initiators and non-initiators were estimated at 12 weeks. In the overall cohort, we found an increased risk of suicidal behaviour among SSRI initiators (intention-to-treat RR = 1.50, 95% CI = 1.25, 1.80; per-protocol RR = 1.69, 95% CI = 1.20, 2.36). In age strata, we only found evidence of an increased risk among individuals under age 25, with the greatest risk among 6–17-year-olds (intention-to-treat RR = 2.90, 95% CI = 1.72, 4.91; per-protocol RR = 3.34, 95% CI = 1.59, 7.00). Our finding of an increased suicidal behaviour risk among individuals under age 25 reflects evidence from RCTs. We found no evidence of an effect in the high-risk group of individuals with past suicidal behaviour. Further studies with information on a wider array of confounders are called for.

## Introduction

Suicide is a leading cause of mortality worldwide [1]. One of the major risk factors for suicide is mood disorders, for which antidepressant medication is the main pharmacotherapeutic option. Meanwhile, selective serotonin reuptake inhibitors (SSRIs) are the first-line pharmacological treatment for most of these disorders [2, 3].

Despite this, there is long-standing concern that SSRI treatment could in itself raise the risk of suicidal behaviour [4]. There is evidence from randomised controlled trials (RCTs) that antidepressant treatment increases risk of newly onset suicide attempts or ideation in children and adolescents [5]. The evidence among adults is conflicting [6–9], though some evidence suggests a neutral effect on suicidal behaviour [6, 10]. However, individual RCTs are underpowered for rare outcomes such as suicide attempts and deaths [11], and tend to have short follow-ups. Important clinical subgroups—such as individuals with a history of suicide ideation or attempts [12, 13]—have usually been excluded. Observational studies can therefore generate valuable evidence on rare but serious outcomes from populations that are representative of those who receive antidepressants in clinical practice.

A promising approach [14] for observational studies that aim to make causal inferences is to apply the study design principles of randomised trials—that is, to use observational data to emulate the target trial one would ideally conduct. This approach is increasingly used in epidemiology, though to our knowledge has not yet been applied to investigating the impact of SSRI initiation on suicidal behaviour. While emulating a target trial does not remove bias from unmeasured confounding, it enables a structured approach to study design that can minimise other common biases [15]. It also allows for a more transparent reporting of study design and analysis process, which aids interpretation of results [16].

This study, therefore, emulates a target trial to assess how SSRI treatment following a depression diagnosis affects the risk of suicidal behaviour. We also consider the effect in subgroups stratified by age, sex, and a history of suicidal behaviour.

## Materials and methods

We seek to emulate a pragmatic target trial in an observational setting—see Table S1 for details on the target trial and our emulation of it.

### Data sources

We used an administrative health data registry that includes healthcare information on all individuals resident in Stockholm county 2006–2019, amounting to around 3 million people [17]. These records are linked to a range of registers, including: the Prescribed Drug Register for medication information, the VAL database for information on primary and secondary care consultations, the Population Register for information on deaths and causes of death, the Medical Birth Register for information on births, and the longitudinal integrated database for health insurance and labour market studies (LISA) register for information on socioeconomic variables [17].

### The target trial and its emulation

#### Eligibility criteria

**Fig. 1:**
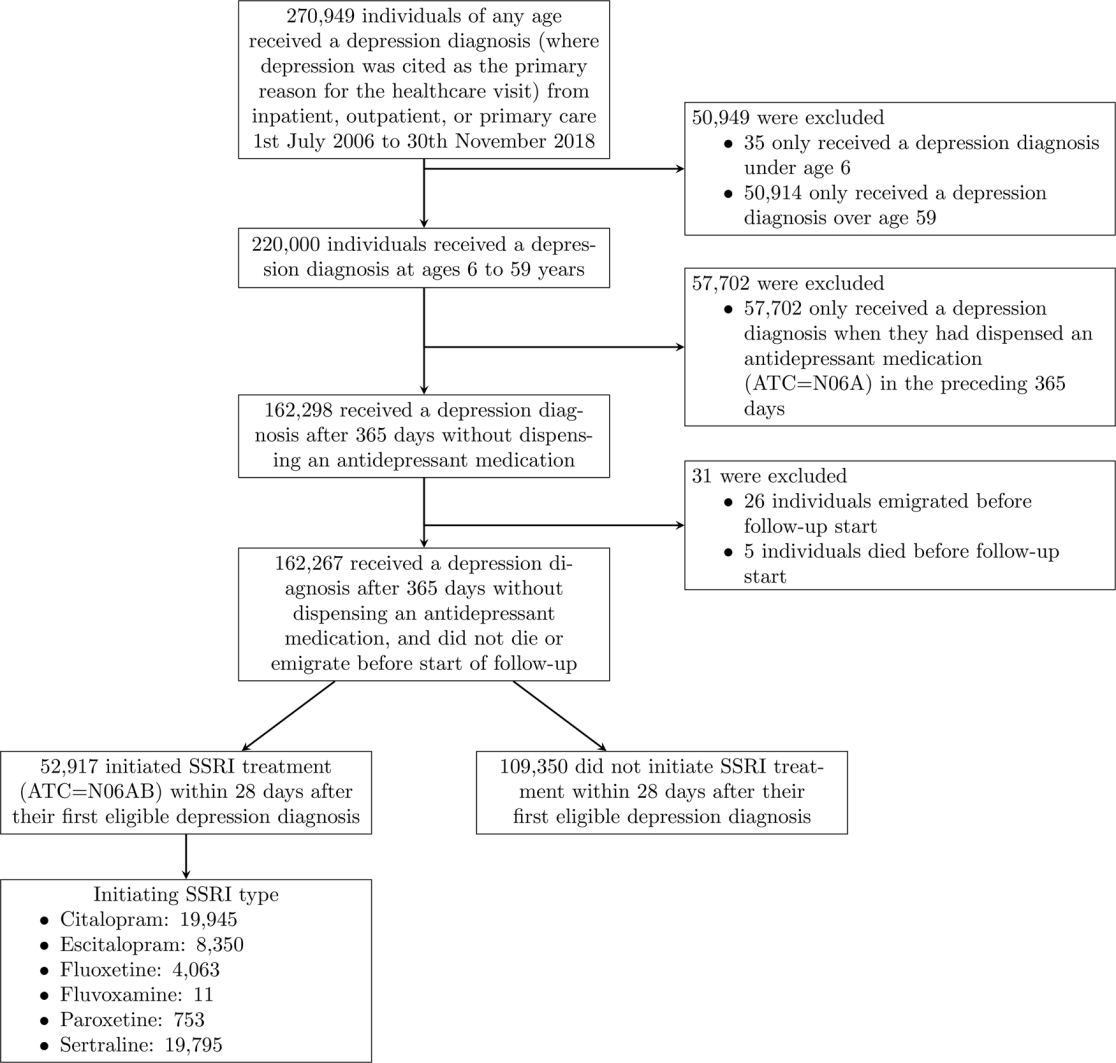
Flow chart of cohort inclusion. Flow chart of cohort inclusion, with reasons for exclusions at each stage.

We selected individuals who received a depression diagnosis (ICD10 = F32-F33) from ages 6 to 59 years during 1st July 2006–30th November 2018, after at least 365 days without antidepressant (ATC = N06A) dispensation. We only included the first recorded eligible depression diagnosis for each individual, where the depression diagnosis was the main reason for the healthcare contact. Start of follow-up is described below; those who died or emigrated between diagnosis and start of follow-up were excluded. See Fig. 1 for a flowchart of study inclusion.

#### Treatment strategies

The treatment strategies were: (1) initiation of any SSRI (N06AB) within 28 days of depression diagnosis then continuing to take it for 12 weeks without ending treatment; and (2) no initiation of any SSRI within 28 days of depression diagnosis and then remaining off treatment for 12 weeks.

#### Treatment assignment

We defined individuals who initiated an SSRI within 28 days as “initiators”, and individuals who did not as “non-initiators”. We assumed that individuals were randomly assigned to their treatment strategies at the start of follow-up, within levels of the baseline covariates. These were: sex, age category, subtype of depression diagnosis, source of depression diagnosis, highest level of attained education of individual, max attained education in the household, family income category, diagnoses (bipolar disorder, anxiety, ADHD, schizophrenia, substance use disorder (excluding alcohol), alcohol use disorder, autism spectrum disorder, history of suicide attempt), medication receipt within last 3 months (antipsychotics, hypnotics and sedatives excluding benzodiazepines, benzodiazepines, antiepileptics, ADHD medication), and year of diagnosis. See Table 1, Table S2, and Table S3 for levels and definitions of the different covariates.

**Table 1:** Demographics.

	Before IP weighting			After IP weighting		
	Initiator	Non-initiator	SMD^a^	Initiator	Non-initiator	SMD^a^
*N*	52917	109350		51676.8	109591.6	
Female (%)	34213 (64.7)	68759 (62.9)	0.037	32745.4 (63.4)	69438.3 (63.4)	<0.001
Age category (years) (%)			0.229			0.039
6–17	1760 (3.3)	9162 (8.4)		2966.9 (5.7)	7319.5 (6.7)	
18–24	8560 (16.2)	18107 (16.6)		8532.3 (16.5)	17869.7 (16.3)	
25–39	21438 (40.5)	44275 (40.5)		21029.3 (40.7)	44310.2 (40.4)	
40–49	12143 (22.9)	21840 (20.0)		10991.5 (21.3)	23067.4 (21.0)	
50–59	9016 (17.0)	15966 (14.6)		8156.9 (15.8)	17024.8 (15.5)	
Family income category (%)			0.203			0.037
<0	64 (0.1)	158 (0.1)		71.9 (0.1)	150.0 (0.1)	
0	460 (0.9)	1130 (1.0)		503.6 (1.0)	1070.1 (1.0)	
0 < x <= 20th percentile	10882 (20.6)	26360 (24.1)		11843.4 (22.9)	25132.0 (22.9)	
20th percentile < x <= 80th percentile	33291 (62.9)	64047 (58.6)		31259.1 (60.5)	65724.1 (60.0)	
>80th percentile	7203 (13.6)	12217 (11.2)		6308.8 (12.2)	13178.4 (12.0)	
NA	1017 (1.9)	5438 (5.0)		1690.0 (3.3)	4337.0 (4.0)	
Education category (%)			0.192			0.031
Primary	9133 (17.3)	20362 (18.6)		9429.1 (18.2)	19896.3 (18.2)	
Secondary	21370 (40.4)	39946 (36.5)		19725.9 (38.2)	41447.8 (37.8)	
Post-secondary	20385 (38.5)	40169 (36.7)		19458.1 (37.7)	40918.6 (37.3)	
NA	2029 (3.8)	8873 (8.1)		3063.7 (5.9)	7328.8 (6.7)	
Family education category (%)			0.178			0.029
Primary	4282 (8.1)	9588 (8.8)		4436.3 (8.6)	9382.0 (8.6)	
Secondary	20075 (37.9)	38437 (35.2)		18843.4 (36.5)	39588.1 (36.1)	
Post-secondary	26698 (50.5)	53186 (48.6)		25579.1 (49.5)	53893.1 (49.2)	
NA	1862 (3.5)	8139 (7.4)		2817.9 (5.5)	6728.4 (6.1)	
Source of depression diagnosis (%)			0.288			0.033
Primary care	41205 (77.9)	71257 (65.2)		36620.8 (70.9)	76039.7 (69.4)	
Outpatient care	10761 (20.3)	35847 (32.8)		14076.6 (27.2)	31419.2 (28.7)	
Inpatient care	951 (1.8)	2246 (2.1)		979.3 (1.9)	2132.8 (1.9)	
Bipolar disorder diagnosis (%)	272 (0.5)	1390 (1.3)	0.081	464.7 (0.9)	1126.6 (1.0)	0.013
Anxiety disorder diagnosis (%)	22775 (43.0)	46992 (43.0)	0.001	22692.4 (43.9)	47536.2 (43.4)	0.011
Schizophrenia diagnosis (%)	414 (0.8)	1599 (1.5)	0.065	628.4 (1.2)	1375.8 (1.3)	0.004
Alcohol use disorder diagnosis (%)	3132 (5.9)	6563 (6.0)	0.004	3175.0 (6.1)	6635.1 (6.1)	0.004
Substance abuse disorder (excl. alcohol) diagnosis (%)	2376 (4.5)	5477 (5.0)	0.024	2599.3 (5.0)	5404.9 (4.9)	0.005
ADHD diagnosis (%)	1104 (2.1)	3580 (3.3)	0.074	1489.0 (2.9)	3183.3 (2.9)	0.001
Autism spectrum disorder diagnosis (%)	387 (0.7)	1309 (1.2)	0.048	560.9 (1.1)	1160.0 (1.1)	0.003
History of suicidal behaviour (%)	1256 (2.4)	2965 (2.7)	0.021	1353.8 (2.6)	2867.5 (2.6)	<0.001
Antipsychotic medication (%)	785 (1.5)	2189 (2.0)	0.040	1032.1 (2.0)	2069.4 (1.9)	0.008
Hypnotics and sedatives medication (%)	22758 (43.0)	23740 (21.7)	0.467	15464.1 (29.9)	31919.8 (29.1)	0.018
Benzodiazepine medication (%)	7298 (13.8)	5681 (5.2)	0.296	4393.7 (8.5)	9141.1 (8.3)	0.006
Antiepileptic medication (%)	635 (1.2)	2002 (1.8)	0.052	849.1 (1.6)	1811.1 (1.7)	0.001
ADHD medication (%)	398 (0.8)	1513 (1.4)	0.061	616.8 (1.2)	1306.2 (1.2)	<0.001

^a^Covariate balance between initiators and non-initiators before and after inverse probability weighting at baseline.

^a^*SMD* Standardised Mean Difference.

#### Outcomes

Our primary endpoint was suicidal behaviour within 12 weeks after baseline, which included hospital visits (outpatient attendance or inpatient admission) for suicide attempts, and deaths from suicide. In line with previous studies [18], we included events with both known intent (ICD-10 codes X60-X84) and unknown intent (ICD-10 codes Y10-Y34).

#### Follow-up

Follow-up start (baseline) was defined as the date when an individual collected (dispensed) their initiating SSRI prescription among initiators; among non-initiators, the start of follow-up was frequency matched from initiators based on the number of days between the depression diagnosis and the initiation of SSRI treatment [19]. We choose to start follow-up at SSRI initiation among initiators, and frequency match among controls to mitigate the impact of immortal time bias [19]. Only 5 deaths, of which 2 by suicide, occurred between fulfilment of study eligibility and start of follow-up among potential study participants (Fig. 1). In order to further investigate whether our results were affected by immortal time bias, we also carried out an analysis using the cloning-censoring-weighting approach [20]—see the section on sensitivity analyses. Follow-up continued for 12 weeks after baseline, or until: death from non-suicide causes, emigration, occurrence of the outcome, administrative end of follow-up, whichever occurred earliest. In the per-protocol analyses, we additionally censored individuals when they stopped adhering to their assigned treatment strategy. As a secondary analysis, we allowed for up to 52 weeks of follow-up.

In the per-protocol analysis, a continuous treatment period with an SSRI was defined based on the assumption that two dispenses falling within 120 days (4 months) of each other belong to the same treatment period—the treatment periods were defined independently of the study follow-up [21]. At the last or single dispensation in a treatment period, the treatment end was defined by adding the population average number of days between consecutive dispenses for the specific medication type to the date of dispensation. This definition is used based on prior work [22]. In all analyses, we estimated treatment periods for the time-varying psychotropic treatment covariates in the same way as the primary way of defining treatment periods for SSRIs (the 4-month approach).

#### Causal contrast

We estimated intention-to-treat and per-protocol effects in our emulated target trial. The intention-to-treat effect is the effect of being assigned to initiate an SSRI vs. not to initiate an SSRI, where initiation is defined as dispensing an SSRI prescription. The per-protocol effect is the effect of being assigned to and fully adhering to the treatment strategy as specified in the protocol.

### Statistical analysis

We used the standardised mean difference (SMD) between initiators and non-initiators to quantify the balance of measured covariates at baseline between the treatment groups—before and after weighting. An SMD of 0.1 or lower is taken as evidence of sufficient covariate balance between groups [23]. Using pooled logistic regression models with product terms between treatment and time [24], we estimated the cumulative incidence (risk), risk difference (RD), and risk ratio (RR) of suicidal behaviour at 12 weeks for all causal contrasts. As a secondary analysis, we also estimated them at 52 weeks. We applied inverse probability weighting (IPW) to adjust for baseline confounders in all analyses [25]. In per-protocol analyses, we additionally censored individuals when they ceased adhering to their assigned treatment strategy. Among SSRI initiators, the end of adherence occurred if the SSRI treatment period ended within <12 weeks. Among individuals assigned as non-initiators, the end of treatment adherence was the date they initiated an SSRI medication, if applicable. We assumed that individuals were randomly censored due to non-adherence in each week of follow-up within levels of the baseline and time-varying confounders. The time-varying confounders were time-varying treatment with: non-SSRI antidepressants, benzodiazepines, and any other psychotropic drug over the follow-up (see Table S3 for variable definitions). All time-varying confounders were updated weekly. We estimated time-varying treatment adherence weights, which took into account both baseline and time-varying confounders. We weighted each individual at each week of follow-up by the product of the baseline IPW weights and the time-varying adherence weights. All weights were stabilised, and truncated at the 99th percentile in order to make sure extreme observations did not make outsize contributions to results. We presented the distribution of weights before and after truncation. 95% confidence intervals were calculated by using non-parametric bootstraps over 500 samples. See supplementary methods for details on the approach. We presented inverse probability weighted cumulative risk curves for the main intention-to-treat analyses. We presented numbers needed to harm (NNH) for the per-protocol analyses over 12 weeks’ follow-up, overall and in the two youngest strata where we found a statistically significant effect. We also calculated the *E*-value for the per-protocol risk ratio over 12 weeks’ follow-up (main analysis), in order to quantify the amount of unmeasured confounding necessary to negate any associations found [26].

### Sensitivity analyses

We conducted several sensitivity analyses to assess the robustness of our results.

We conducted three analyses where we redefined treatment strategies (“initiation of any SSRI within 28 days of depression diagnosis”/“no initiation of an SSRI within 28 days of depression diagnosis”) as initiation/no initiation of any SSRI within 7, 14, or 84 days of depression diagnosis, respectively.

We also considered analyses where a cloning-censoring-weighting set-up was employed to define start of follow-up. In this analysis, a clone of each individual was entered into each treatment strategy arms (“initiation of any SSRI within 28 days of depression diagnosis”/“no initiation of an SSRI within 28 days of depression diagnosis”) at the date of depression diagnosis. If and when an individual initiated an SSRI during the grace period, the clone in the non-initiator arm was censored. If an individual did not initiate an SSRI during the grace period, a clone of the individual remained in each arm until 28 days after the diagnosis, when the clone in the “SSRI initiation”-arm was censored. Inverse probability weighting was employed to account for censoring [20].

For the outcome definition, we conducted a sensitivity analysis where we only included suicidal behaviour events of known intent (ICD-10 codes X60-X84).

For the treatment period definition, we conducted sensitivity analyses where treatment periods were defined using the assumption that individuals take one pill per day. This is to assess the impact of borrowing information on dispensed prescriptions from the future in the main treatment period definition [27].

For the per-protocol analyses, we carried out a sensitivity analysis where we additionally adjusted for the following diagnoses given during the follow-up as time-varying confounders: bipolar disorder, anxiety disorder, alcohol disorder, substance use disorder, and schizophrenia (See Table S3 for variable definitions).

## Results

We identified 162,298 individuals who fulfilled study eligibility criteria of a depression diagnosis after a 1-year period without antidepressant dispensation. After start of follow-up was defined in the way set out in the methods, 162,267 individuals remained (5 individuals died and 26 emigrated in the 28-day grace period; Fig. 1). Of those, 52,917 initiated an SSRI within 28 days (“initiators”) and 109,350 did not (“non-initiators”). Among SSRI initiators, 20,352 (38%) discontinued their treatment within 12 weeks; among non-initiators, 7965 (7%) initiated SSRIs within 12 weeks.

Table 1 and Table S2 show the baseline covariates and their proportions in initiators and non-initiators before and after applying IPW for baseline covariates. After IPW weighting, the SMD was below 0.1 for all covariates. Table S4 shows the distribution of weights before and after truncation for the analyses over 12 weeks.

**Fig. 2:**
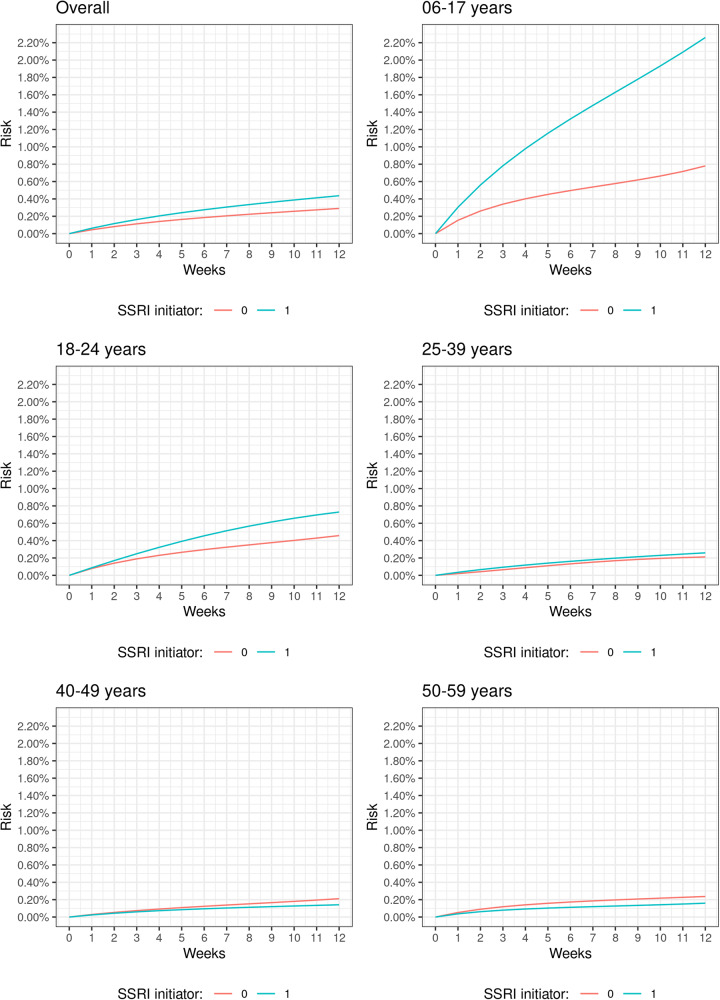
Intention-to-treat risk curves over 12 weeks in SSRI initiators and non-initiators, overall and stratified by age. Each panel of the figure represents a different age stratum, with the first panel representing the overall population. The risk curves for SSRI initiators are given in blue and those for non-initiators in red.

Table 2 shows the findings from the intention-to-treat and per-protocol analyses that consider a follow-up of 12 weeks. The absolute risk among SSRI initiators was greater than among non-initiators in the overall cohort, with intention-to-treat absolute risks of 0.44% (95% CI = 0.37%, 0.50%) among initiators and 0.29% (95% CI = 0.26%, 0.32%) among non-initiators, corresponding to RDs and RRs of 0.15% (95% CI = 0.07%, 0.22%) and 1.50 (95% CI = 1.25, 1.80), respectively. Figure 2 shows the intention-to-treat cumulative risk curves over 12 weeks, overall and stratified by age.

**Table 2:** Intention-to-treat and per-protocol analyses over 12 weeks, overall and by age.

	N events, initiators	N events, non-initiators	Absolute risk, initiators % (95% CI)	Absolute risk, non-initiators % (95% CI)	Risk difference % (95% CI)	Risk ratio (95% CI)
*Intention-to-treat*^*a*^						
Overall	207	323	0.44 (0.37,0.50)	0.29 (0.26,0.32)	0.15 (0.07,0.22)	1.50 (1.25,1.80)
6–17 years	47	67	2.26 (1.04,3.48)	0.78 (0.58,0.97)	1.48 (0.26,2.71)	2.90 (1.72,4.91)
18–24 years	76	76	0.73 (0.54,0.92)	0.46 (0.34,0.57)	0.27 (0.05,0.49)	1.59 (1.11,2.28)
25–39 years	54	94	0.26 (0.19,0.33)	0.21 (0.17,0.25)	0.05 (−0.04,0.13)	1.22 (0.86,1.73)
40–49 years	18	45	0.14 (0.07,0.21)	0.21 (0.14,0.28)	−0.07 (−0.17,0.03)	0.67 (0.36,1.25)
50–59 years	12	41	0.16 (0.04,0.28)	0.24 (0.16,0.31	−0.08 (−0.21,0.06)	0.67 (0.31,1.45)
*Per-protocol*^*b*^						
Overall	188	305	0.47 (0.34,0.60)	0.28 (0.23,0.34)	0.19 (0.05,0.33)	1.69 (1.20,2.36)
6–17 years	45	61	2.41 (0.92,3.90)	0.72 (0.41,1.04)	1.69 (0.17,3.20)	3.34 (1.59,7.00)
18–24 years	72	70	0.85 (0.50,1.21)	0.42 (0.25,0.60)	0.43 (0.03,0.83)	2.01 (1.12,3.60)
25–39 years	48	93	0.27 (0.13,0.40)	0.21 (0.14,0.29)	0.05 (−0.11,0.21)	1.24 (0.65,2.35)
40–49 years	15	42	0.14 (0.03,0.25)	0.20 (0.09,0.31)	−0.07 (−0.22,0.09)	0.68 (0.25,1.84)
50–59 years	8	39	0.12 (−0.02,0.26)	0.23 (0.10,0.36)	−0.11 (−0.30,0.08)	0.53 (0.14,2.04)

^a^IPW weighted for baseline treatment assignment using baseline confounders.

^b^IPW weights for baseline treatment assignment (using baseline confounders only) are multiplied by time-varying weights for weighting by treatment adherence (using baseline confounders and time-varying indicators of treatment with other medications).

The per-protocol absolute risks were 0.47% (95% CI = 0.34%, 0.60%) among initiators and 0.28% (95% CI = 0.23%, 0.34%) among non-initiators, corresponding to an RD of 0.19% (95% CI = 0.05%, 0.33%), an RR of 1.69 (95% CI = 1.20, 2.36), and an NNH of 526. For the per-protocol RR in the overall cohort, the *E*-value was 2.77 for the effect estimate, and 1.69 for the lower confidence interval.

When stratifying on age, we find the greatest intention-to-treat effect estimates among 6–17-year-olds (RD = 1.48%, 95% CI = 0.26%, 2.71%; RR = 2.90, 95% CI = 1.72, 4.91), followed by 18–24-year-olds (RD = 0.27%, 95% CI = 0.05%, 0.49%; RR = 1.59, 95% CI = 1.11, 2.28). There was no evidence of an effect in individuals aged 25 and above from intention-to-treat analyses, with RDs of 0.05% (95% CI = −0.04%, 0.13%), −0.07% (95% CI = −0.17%, 0.03%), and −0.08% (95% CI = −0.21%, 0.06%); and RRs of 1.22 (95% CI = 0.86,1.73), 0.67 (95% CI = 0.36,1.25), and 0.67 (95% CI = 0.31,1.45) for 25–39-year-olds, 40–49-year-olds, and 50–59-year-olds, respectively.

We find similar age patterns for the per-protocol analysis. The greatest per-protocol effect estimates were among 6–17-year-olds (RD = 1.69%, 95% CI = 0.17%, 3.20%; RR = 3.34, 95% CI = 1.59, 7.00; NNH = 59), followed by 18–24-year-olds (RD = 0.43%, 95% CI = 0.03%, 0.83%; RR = 2.01, 95% CI = 1.12, 3.60; NNH = 233). There was no evidence of a per-protocol effect in individuals aged 25 and above, with RDs of 0.05% (95% CI = −0.11%,0.21%), −0.07% (95% CI = −0.22%, 0.09%), and −0.11% (95% CI = −0.30%,0.08%); and RRs of 1.24 (95% CI = 0.65,2.35), 0.68 (95% CI = 0.25,1.84), and 0.53 (95% CI = 0.14,2.04) for 25–39-year-olds, 40–49-year-olds, and 50–59-year-olds, respectively.

Those with no history of suicidal behaviour (*N* = 158,046) had a similar, though slightly elevated, per-protocol RR as compared to the overall cohort (Table 3), though the absolute risks and RD were very similar to those in the overall cohort. Meanwhile, those with a history of suicidal behaviour (*N* = 4221) showed greater absolute risks among both initiators and non-initiators. We found no evidence of a difference between initiators and non-initiators in this group in terms of suicidal behaviour risk (RD = 0.42%, 95% CI = −1.49%, 2.34%; RR = 1.17, 95% CI = 0.58, 2.34).

**Table 3:** Per-protocol analyses over 12 weeks, stratified by history of suicidal behaviour at start of follow-up.

History of suicidal behaviour (yes/no)	N events, initiators	N events, non-initiators	Absolute risk, initiators % (95% CI)	Absolute risk, non-initiators % (95% CI)	Risk difference % (95% CI)	Risk ratio (95% CI)
No^a^	155	227	0.41 (0.28,0.53)	0.22 (0.17,0.27)	0.19 (0.06,0.32)	1.87 (1.28,2.72)
Yes^b^	33	78	2.94 (1.22,4.66)	2.52 (1.59,3.44)	0.42 (−1.49,2.34)	1.17 (0.58,2.34)

^a^N individuals = 158,046.

^b^N individuals = 4221.

In the sex-stratified analyses (Table S5), there was a greater RR of suicidal behaviour among females as compared to males in the overall cohort (RR = 1.91 vs. 1.38). Among males, data were not compatible with a difference in suicidal behaviour risk between SSRI initiators and non-initiators. The age-stratified results in females were similar to those in the overall cohort, apart from among those aged 50–59 years, where only one event occurred among SSRI initiators. Among males, the youngest age category had a lower RR and risk difference compared to the 6–17-year-olds in the overall population (RD = 0.37% vs. 1.69%, RR = 1.68 vs. 3.34); in the remaining age groups, the age pattern of results among males was similar to that in the overall cohort.

Table S6 shows the intention-to-treat and per-protocol analysis results over 52 weeks in the overall cohort. As compared to the estimates over 12 weeks’ follow-up, the 52-week follow-up showed a lower RR for the intention-to-treat analysis (1.39 vs. 1.50) but a higher RR for the per-protocol analysis (1.93 vs. 1.69). Figure S1 shows the intention-to-treat cumulative risk curve over 52 weeks.

When considering only suicidal behaviour events of known intent as the outcome in the per-protocol analysis over 12 weeks’ follow-up (Table S7), results were similar to the main analysis. When creating treatment periods using the assumption that individuals take 1 SSRI pill per day, effect estimates were similar though slightly attenuated as compared to the main analysis (Table S7). Accounting for a range of time-varying diagnoses in the per-protocol weights (Table S7) had virtually no impact on results as compared to the main analysis.

We explored the impact of changing the period allowed between the eligible depression diagnosis and assignment of SSRI initiation or non-initiation to 7, 14, or 84 days (Table S8). Findings were similar to the main results in all these scenarios. The 7- and 14-day grace periods led to unchanged absolute risks among non-initiators, but a somewhat lower absolute risk among initiators led to slightly reduced RR point estimates. The 84-day grace period led to unchanged absolute rates in the SSRI initiators, but a lower absolute rate among non-initiators led to a higher RR, albeit very similar risk differences, compared to the main analysis.

Finally, we employed a cloning-censoring-weighting approach to assess whether our results were impacted by immortal time bias (Table S9). In this set-up, all 162,298 individuals with a depression diagnosis after 365 days of no antidepressant receipt were included. The results were similar to the main analyses.

## Discussion

In this cohort of 162,267 individuals, we found that there was a higher risk of suicidal behaviour among individuals who initiated an SSRI within 28 days after a depression diagnosis than among those who did not. When stratifying on age, we only found evidence of an increased risk in the two youngest age categories—the intention-to-treat RR was 2.90 (95% CI = 1.72, 4.91) and 1.59 (95% CI = 1.11, 2.28); and the per-protocol RR was 3.34 (95% CI = 1.59, 7.00), and 2.01 (95% CI = 1.12, 3.60) among 6–17- and 18–24-year-olds, respectively. The absolute risk was elevated among individuals with a history of suicidal behaviour, where we found no evidence of a difference in the risk of suicidal behaviour between SSRI initiators and non-initiators.

The present study finds similar results to prior observational research—that is, consistent evidence of an increased risk of suicidality during treatment with SSRIs in children and adolescents [28, 29]. In adults, several studies find an unchanged or lower risk of suicidality during treatment with SSRIs [29] while others find SSRI-treated periods to carry an increased risk [30, 31]. A recent study on the impact of SSRI initiation in a Swedish register setting, utilising a within-individual design, found an elevated risk of suicidal behaviour in the first year of SSRI treatment as compared to the month a year prior to SSRI initiation across age groups, but a reduced risk when comparing the month immediately after to immediately before initiation [22]. While that paper accounted for all time-invariant confounding within-individuals, it could neither control for time-varying confounding by the course of the disorder indicating an individual for treatment, nor for the impact of contact with the healthcare service, which receipt of an SSRI is a proxy for. By comparison, while the current study is subject to between-individual confounding, it minimises other common sources of bias, such as reverse causation bias [14] by emulating a target trial. It also provides a structured and clinically useful research question.

Similar to the findings reported here, RCTs have also consistently found an increased risk of suicidal behaviour in antidepressant arms among children and adolescents [6, 10, 32], while several find no evidence of an effect in adults [6, 10]. The similarity of the results in this observational cohort to those from RCTs lends more confidence to the interpretation of results from subgroups that have not been studied in an RCT setting.

In particular, RCTs to date have routinely excluded individuals with a history of suicidal behaviour, despite observational evidence that prior suicide attempts is a major predictor of subsequent suicidal behaviour [1, 33]. When we stratify on past suicidal behaviour, we find that absolute risk of suicidal behaviour is elevated, regardless of whether an individual initiated an SSRI or not. This is in line with previous research in Swedish register data [22]. However, in this high-risk subgroup, there was no evidence for a difference in suicidal behaviour risk between SSRI initiators and non-initiators. This may be due to several reasons: that this stratification more fully accounts for unmeasured confounding by depression severity (i.e. confounding by indication); that prescribers follow a different clinical decision-making process in these individuals, meaning individuals with particularly high risk could be selected into the non-initiator group, masking a causal effect of SSRIs on suicidal behaviour; that there is a different risk profile of SSRIs in this subgroup; or that we have a lack of statistical power—only 4221 individuals in our cohort had a history of suicidal behaviour. Further research is necessary to investigate the effects of SSRI treatment in this subgroup.

Another issue with extant RCTs is that they generally have short follow-up—often in the span of days or a few weeks—which makes it challenging to determine how the risk of suicide changes over treatment time. However, our analysis of a 52-week follow-up showed no major difference in results as compared to the 12-week follow-up.

A key issue with interpreting our results is the likely impact of confounding by indication [29]. In particular, we did not have information on depression severity: it is possible that those with more severe depression are more likely to be prescribed an SSRI medication, which could explain the elevated risks that we see among SSRI initiators. This possibility is supported by the fact that psychological therapies are the first-line treatment for mild and moderate depression among both children and adults in Sweden [34], meaning that a prescription of an SSRI within a short time after a depression diagnosis may indicate that the underlying depression is severe enough to warrant pharmacological intervention. This is more likely among children and adolescents, where there are established concerns regarding initiating SSRI treatment [35]. We also could not account for other factors influencing medication receipt, such as personal preference, and did not have information on other types of treatment individuals may have accessed, including psychotherapy. It should further be noted that our findings are at the group level—it is possible that a minority of individuals with specific clinical characteristics are at increased risk of suicidal behaviour during SSRI treatment. While we have attempted to investigate important clinical subgroups, such as individuals with a history of suicidal behaviour, there may be others—including cases where many characteristics interact in complex ways to influence the risk of suicidal behaviour during SSRI treatment.

Nevertheless, our results confirm that children and adolescents under age 25 are a high-risk group, in particular children aged under 18 years. Children and adolescents have been the focus of concerns regarding SSRI treatment, with the US Food and Drug Administration Black Box warning specific to individuals under age 25 [35]. Our results do not support an increased risk among older adults, which has also been a debated area [9, 36]. Our findings further highlight that individuals with a history of suicidal behaviour have an elevated risk of further suicidal behaviour as compared to individuals with no such history, which corresponds to prior evidence [1, 22, 33]. While we find no evidence of an effect of SSRI treatment on suicidal behaviour in this group, further research is called for.

### Strengths and limitations

Our results draw on a cohort representative of the entire population of Stockholm county 2006–2019, ensuring a relatively large number of SSRI initiators. These records were linked to routinely collected Swedish register data, ensuring a broad array of available covariates with a high level of validity. We have sought to avoid common biases in epidemiological studies by emulating a target trial [37].

However, our study also suffers from several limitations. First, as discussed, our analyses could not account for unmeasured confounding, including confounding by indication. Our *E*-value of 2.77 for the effect estimate, and 1.69 for the lower confidence interval, in the overall 12-week per-protocol analysis indicates that a moderate amount of unmeasured confounding would be sufficient to produce our results, given a true null effect [26]. Second, we did not consider risks within shorter time intervals than weeks, such as days after initiation. There is evidence that individuals may experience fast onset of suicidal behaviour after initiating SSRI treatment [4]. Third, we could not consider suicidal ideation, despite the importance of this outcome in RCTs. Similarly, we were unable to include suicide attempts that did not result in contact with the healthcare service. Still, our outcome is likely to include the more severe suicidal behaviour events that are most important to prevent. Fourth, our results are mainly relevant to suicide attempt—death by suicide was very rare in our cohort. Fifth, our use of a 28-day grace period meant that some individuals were censored by death or emigration before they could enter the cohort, but this occurrence was very rare, and our results were unchanged when reducing the grace period to 7 days. Results were also largely unchanged when we employed a cloning-censoring-weighting approach. Sixth, we could not determine whether individuals consumed purchased medication, although this is an issue in any pharmacoepidemiology study. Similarly, our treatment period definition may lead to misclassification of follow-up time, though using an alternative method to define treatment periods did not substantially affect results, and this limitation only applied to per-protocol analyses.

## Conclusion

We found an increased risk of suicidal behaviour among individuals aged below 25 years who were treated with an SSRI after a depression diagnosis as compared to those who were not. We found no evidence of an effect among older age categories. The results are similar to those from RCTs, lending validity to this observational study. However, the issue of unmeasured confounding remains, and studies from data sources with more detailed information on confounders—notably depression severity—are called for. Our results confirm that individuals with a history of suicidal behaviour are a high-risk group, but we found no evidence that SSRI initiation conferred elevated risks of suicidal behaviour in this subgroup. The effect of SSRI treatment on suicidal behaviour among those with a past history of this outcome requires further investigation, especially as this subgroup has not been sufficiently studied in RCTs.

### Supplementary information

Supplemental material
