# Supplementary material for: Effect of selective serotonin reuptake inhibitor treatment following diagnosis of depression on suicidal behaviour risk: a target trial emulation
Source: Neuropsychopharmacology. 2023 Jul 28;48(12):1760–8. doi: 10.1038/s41386-023-01676-3 (PMC10579366; doi:10.1038/s41386-023-01676-3)
Supplement: Supplementary file 1 — Supplemental material [file 41386_2023_1676_MOESM1_ESM.docx]

**Supplement**

**Contents**

[**Table S1. Target trial emulation** 2](#_Toc141305272)

[**Table S2. Demographics – full table** 4](#_Toc141305273)

[**Table S3. Codes for diagnosis and medication covariates** 6](#_Toc141305274)

[**Table S4. Distribution of weights in analyses with 12 weeks’ follow-up, before and after truncation** 7](#_Toc141305275)

[**Table S5. Per-protocol analyses over 12 weeks, stratified by sex and age** 7](#_Toc141305276)

[**Table S6. Intention-to-treat and per-protocol analyses over 52 weeks** 8](#_Toc141305277)

[**Table S7. Per-protocol analyses over 12 weeks, with alternative definition of events, alternative definition of treatment period length, or additional time-varying variables** 8](#_Toc141305278)

[**Table S8. Per-protocol analyses over 12 weeks using alternative grace periods** 9](#_Toc141305279)

[**Table S9. Intention-to-treat and per-protocol analyses using the cloning-censoring-weighting approach** 9](#_Toc141305280)

[**Figure S1. Intention-to-treat risk curves over 52 weeks in initiators and non-initiators** 10](#_Toc141305281)

[**Supplementary methods** 11](#_Toc141305282)

## **Table S1. Target trial emulation**

| Component | Target trial specification | Target trial emulation |
| --- | --- | --- |
| **Eligibility criteria** | - Age 6-59 years old 1^st^ July 2006 – 30^th^ November 2018 - First diagnosis of depression (ICD-10: F32-F33) 1^st^ July 2006 to 30^th^ November 2018 - No antidepressant (N06A) prescription dispensations within 365 days before depression diagnosis | Same as target trial. |
| **Treatment strategies** | 1. Initiation of any SSRI (N06AB) within 28 days of depression diagnosis then continuing to take it for 12 weeks 2. No initiation of any SSRI within 28 days of depression diagnosis and then remaining off treatment for 12 weeks | Same as target trial.    Follow-up starts at time of prescription dispensation in initiators. The number of days between depression diagnosis and prescription dispensation in initiators is randomly assigned to non-initiators – the non-initiators start follow-up on this number of days after their depression diagnosis.^1^  In sensitivity analyses, we consider SSRI initiation within 7, 14, or 84 days.  In further sensitivity analyses, we consider the cloning-censoring-weighting approach for defining start of follow-up, where follow-up starts at time of depression diagnosis receipt for all study participants. |
| **Treatment assignment** | Individuals are randomly assigned to a strategy at baseline and will be aware of the strategy to which they have been assigned. | We classified individuals according to the strategy they are compatible with at baseline.  Participants are assumed to be randomly assigned to treatment at baseline within levels of:  sex, age category, subtype of depression diagnosis, source of depression diagnosis (primary, inpatient, outpatient care), max attained education of individual, max attained education in the household, family income category, history of suicide attempt, diagnoses (bipolar disorder, anxiety, ADHD, schizophrenia, substance use disorder excluding alcohol, alcohol use disorder, autism spectrum disorder), medication receipt within last 3 months (antipsychotics, hypnotics and sedatives excluding benzodiazepines, benzodiazepines, antiepileptics, ADHD medication), year of diagnosis |
| **Outcomes** | Suicidal behaviour: suicide attempts or death from inpatient care or unplanned outpatient care  Intentional: ICD-10 X60 - X84;  Unknown intent: ICD-10 Y10 - Y34 | Same as for target trial. |
| **Follow-up** | Starts at randomization and ends at (whichever occurs first):   - 12 weeks after randomization - Death from non-suicide causes - Emigration - Outcome | Same as for target trial.  In sensitivity analyses, individuals are followed up for 52 weeks after baseline unless they are censored by the other listed events. |
| **Causal contrasts** | 1. Intention-to-treat effect 2. Per-protocol effect | Observational analog of intention-to-treat and per-protocol effect |
| **Analysis plan** | **ITT:** Individuals remain on treatment they are assigned to throughout follow-up  **Per-protocol:** Accounting for fact that individuals may stop adhering to assigned treatment. Individuals will be censored when they cease adhering to their assigned treatment. Bias arising from this censoring will be handled by IPW weighting for adherence to treatment using baseline confounders and time-varying treatment with non-SSRI antidepressants, benzodiazepines, and any other psychotropic drug over follow-up.  Subgroup analyses by baseline age, sex, and history of suicidal behaviour  Age categories: 6-17-year-olds, 18-24-year-olds, 25-39-year-olds, 40-49-year-olds, and 50-59-year-olds | Same intention-to-treat and per-protocol analyses with sequential emulation and additional adjustment for baseline covariates.  Same subgroup analyses. |

## **Table S2. Demographics – full table**

|  | **Before IP weighting** | | | **After IP weighting** | | |
| --- | --- | --- | --- | --- | --- | --- |
|  | **Initiator** | **Non-initiator** | **SMD^a^** | **Initiator** | **Non-initiator** | **SMD^a^** |
| **N** | 52917 | 109350 |  | 51676.8 | 109591.6 |  |
| **Female (%)** | 34213 (64.7) | 68759 (62.9) | 0.037 | 32745.4 (63.4) | 69438.3 (63.4) | <0.001 |
| **Age category (years) (%)** |  |  | 0.229 |  |  | 0.039 |
| 6-17 | 1760 (3.3) | 9162 (8.4) |  | 2966.9 (5.7) | 7319.5 (6.7) |  |
| 18-24 | 8560 (16.2) | 18107 (16.6) |  | 8532.3 (16.5) | 17869.7 (16.3) |  |
| 25-39 | 21438 (40.5) | 44275 (40.5) |  | 21029.3 (40.7) | 44310.2 (40.4) |  |
| 40-49 | 12143 (22.9) | 21840 (20.0) |  | 10991.5 (21.3) | 23067.4 (21.0) |  |
| 50-59 | 9016 (17.0) | 15966 (14.6) |  | 8156.9 (15.8) | 17024.8 (15.5) |  |
| **Year of follow-up start (%)** |  |  | 0.201 |  |  | 0.015 |
| 2006 | 1807 (3.4) | 4781 (4.4) |  | 1983.8 (3.8) | 4437.0 (4.0) |  |
| 2007 | 4209 (8.0) | 6989 (6.4) |  | 3574.7 (6.9) | 7563.0 (6.9) |  |
| 2008 | 4069 (7.7) | 7158 (6.5) |  | 3611.7 (7.0) | 7597.6 (6.9) |  |
| 2009 | 4009 (7.6) | 6850 (6.3) |  | 3511.2 (6.8) | 7363.9 (6.7) |  |
| 2010 | 4503 (8.5) | 7461 (6.8) |  | 3894.3 (7.5) | 8109.3 (7.4) |  |
| 2011 | 4770 (9.0) | 8246 (7.5) |  | 4168.5 (8.1) | 8768.7 (8.0) |  |
| 2012 | 4810 (9.1) | 8676 (7.9) |  | 4350.4 (8.4) | 9117.3 (8.3) |  |
| 2013 | 4637 (8.8) | 9558 (8.7) |  | 4552.5 (8.8) | 9623.6 (8.8) |  |
| 2014 | 4787 (9.0) | 10098 (9.2) |  | 4732.0 (9.2) | 10022.0 (9.1) |  |
| 2015 | 4699 (8.9) | 10328 (9.4) |  | 4724.7 (9.1) | 10082.7 (9.2) |  |
| 2016 | 4025 (7.6) | 9828 (9.0) |  | 4418.0 (8.5) | 9373.9 (8.6) |  |
| 2017 | 3582 (6.8) | 9758 (8.9) |  | 4238.7 (8.2) | 9018.5 (8.2) |  |
| 2018 | 3010 (5.7) | 9619 (8.8) |  | 3916.4 (7.6) | 8514.1 (7.8) |  |
| **Family income category (%)** |  |  | 0.203 |  |  | 0.037 |
| <0 | 64 (0.1) | 158 (0.1) |  | 71.9 (0.1) | 150.0 (0.1) |  |
| 0 | 460 (0.9) | 1130 (1.0) |  | 503.6 (1.0) | 1070.1 (1.0) |  |
| 0 < x <= 20^th^ percentile | 10882 (20.6) | 26360 (24.1) |  | 11843.4 (22.9) | 25132.0 (22.9) |  |
| 20th percentile < x <= 80th percentile | 33291 (62.9) | 64047 (58.6) |  | 31259.1 (60.5) | 65724.1 (60.0) |  |
| >80th percentile | 7203 (13.6) | 12217 (11.2) |  | 6308.8 (12.2) | 13178.4 (12.0) |  |
| NA | 1017 (1.9) | 5438 (5.0) |  | 1690.0 (3.3) | 4337.0 (4.0) |  |
| **Education category (%)** |  |  | 0.192 |  |  | 0.031 |
| Primary | 9133 (17.3) | 20362 (18.6) |  | 9429.1 (18.2) | 19896.3 (18.2) |  |
| Secondary | 21370 (40.4) | 39946 (36.5) |  | 19725.9 (38.2) | 41447.8 (37.8) |  |
| Post-secondary | 20385 (38.5) | 40169 (36.7) |  | 19458.1 (37.7) | 40918.6 (37.3) |  |
| NA | 2029 (3.8) | 8873 (8.1) |  | 3063.7 (5.9) | 7328.8 (6.7) |  |
| **Family education category (%)** |  |  | 0.178 |  |  | 0.029 |
| Primary | 4282 (8.1) | 9588 (8.8) |  | 4436.3 (8.6) | 9382.0 (8.6) |  |
| Secondary | 20075 (37.9) | 38437 (35.2) |  | 18843.4 (36.5) | 39588.1 (36.1) |  |
| Post-secondary | 26698 (50.5) | 53186 (48.6) |  | 25579.1 (49.5) | 53893.1 (49.2) |  |
| NA | 1862 (3.5) | 8139 (7.4) |  | 2817.9 (5.5) | 6728.4 (6.1) |  |
| **Depression diagnosis (%)** |  |  | 0.350 |  |  | 0.075 |
| Depressive episode | 21565 (40.8) | 30967 (28.3) |  | 17261.2 (33.4) | 35622.2 (32.5) |  |
| Mild depressive episode | 3376 (6.4) | 12313 (11.3) |  | 4884.2 (9.5) | 10555.6 (9.6) |  |
| Moderate depressive episode | 7375 (13.9) | 13982 (12.8) |  | 6942.8 (13.4) | 14415.0 (13.2) |  |
| Severe depressive episode, without psychotic symptoms | 1018 (1.9) | 2023 (1.9) |  | 975.2 (1.9) | 2046.8 (1.9) |  |
| Severe depressive episode, with psychotic symptoms | 142 (0.3) | 548 (0.5) |  | 201.6 (0.4) | 464.5 (0.4) |  |
| Other depressive episode | 306 (0.6) | 986 (0.9) |  | 405.3 (0.8) | 870.8 (0.8) |  |
| Unspecified depressive episode | 13983 (26.4) | 33337 (30.5) |  | 14883.1 (28.8) | 31871.1 (29.1) |  |
| Recurrent depressive disorder | 1065 (2.0) | 1614 (1.5) |  | 876.6 (1.7) | 1821.2 (1.7) |  |
| Mild current episode | 607 (1.1) | 2578 (2.4) |  | 969.3 (1.9) | 2142.9 (2.0) |  |
| Moderate current episode | 1776 (3.4) | 4783 (4.4) |  | 2109.0 (4.1) | 4436.8 (4.0) |  |
| Severe current episode, without psychotic symptoms | 290 (0.5) | 799 (0.7) |  | 348.9 (0.7) | 738.1 (0.7) |  |
| Severe current episode, with psychotic symptoms | 47 (0.1) | 176 (0.2) |  | 68.5 (0.1) | 151.0 (0.1) |  |
| Recurrent depressive disorder, currently in remission | 80 (0.2) | 1491 (1.4) |  | 195.5 (0.4) | 1058.7 (1.0) |  |
| Other current episode | 48 (0.1) | 282 (0.3) |  | 83.9 (0.2) | 221.4 (0.2) |  |
| Unspecified current depressive disorder | 1239 (2.3) | 3471 (3.2) |  | 1471.9 (2.8) | 3175.4 (2.9) |  |
| **Source of depression diagnosis (%)** |  |  | 0.288 |  |  | 0.033 |
| Primary care | 41205 (77.9) | 71257 (65.2) |  | 36620.8 (70.9) | 76039.7 (69.4) |  |
| Outpatient care | 10761 (20.3) | 35847 (32.8) |  | 14076.6 (27.2) | 31419.2 (28.7) |  |
| Inpatient care | 951 (1.8) | 2246 (2.1) |  | 979.3 (1.9) | 2132.8 (1.9) |  |
| **Bipolar disorder diagnosis (%)** | 272 (0.5) | 1390 (1.3) | 0.081 | 464.7 (0.9) | 1126.6 (1.0) | 0.013 |
| **Anxiety disorder diagnosis (%)** | 22775 (43.0) | 46992 (43.0) | 0.001 | 22692.4 (43.9) | 47536.2 (43.4) | 0.011 |
| **Schizophrenia diagnosis (%)** | 414 (0.8) | 1599 (1.5) | 0.065 | 628.4 (1.2) | 1375.8 (1.3) | 0.004 |
| **Alcohol use disorder diagnosis (%)** | 3132 (5.9) | 6563 (6.0) | 0.004 | 3175.0 (6.1) | 6635.1 (6.1) | 0.004 |
| **Substance abuse disorder (excl. alcohol) diagnosis (%)** | 2376 (4.5) | 5477 (5.0) | 0.024 | 2599.3 (5.0) | 5404.9 (4.9) | 0.005 |
| **ADHD diagnosis (%)** | 1104 (2.1) | 3580 (3.3) | 0.074 | 1489.0 (2.9) | 3183.3 (2.9) | 0.001 |
| **Autism spectrum disorder diagnosis (%)** | 387 (0.7) | 1309 (1.2) | 0.048 | 560.9 (1.1) | 1160.0 (1.1) | 0.003 |
| **History of suicidal behaviour (%)** | 1256 (2.4) | 2965 (2.7) | 0.021 | 1353.8 (2.6) | 2867.5 (2.6) | <0.001 |
| **Antipsychotic medication (%)** | 785 (1.5) | 2189 (2.0) | 0.040 | 1032.1 (2.0) | 2069.4 (1.9) | 0.008 |
| **Hypnotics and sedatives medication (%)** | 22758 (43.0) | 23740 (21.7) | 0.467 | 15464.1 (29.9) | 31919.8 (29.1) | 0.018 |
| **Benzodiazepine medication (%)** | 7298 (13.8) | 5681 (5.2) | 0.296 | 4393.7 (8.5) | 9141.1 (8.3) | 0.006 |
| **Antiepileptic medication (%)** | 635 (1.2) | 2002 (1.8) | 0.052 | 849.1 (1.6) | 1811.1 (1.7) | 0.001 |
| **ADHD medication (%)** | 398 (0.8) | 1513 (1.4) | 0.061 | 616.8 (1.2) | 1306.2 (1.2) | <0.001 |
| ^a^ SMD = Standardized Mean Difference | | | | | | |
|  |  |  |  |  |  |  |

## **Table S3. Codes for diagnosis and medication covariates**

| **Type** | **Confounder** | **ICD10 / ATC code** |
| --- | --- | --- |
| **Baseline confounders** | **Depression diagnosis** |  |
|  | Depressive episode | F32 |
|  | Mild depressive episode | F32.0 |
|  | Moderate depressive episode | F32.1 |
|  | Severe depressive episode, without psychotic symptoms | F32.2 |
|  | Severe depressive episode, with psychotic symptoms | F32.3 |
|  | Other depressive episode | F32.8 |
|  | Unspecified depressive episode | F32.9 |
|  | Recurrent depressive disorder | F33 |
|  | Mild current episode | F33.0 |
|  | Moderate current episode | F33.1 |
|  | Severe current episode, without psychotic symptoms | F33.2 |
|  | Severe current episode, with psychotic symptoms | F33.3 |
|  | Recurrent depressive disorder, currently in remission | F33.4 |
|  | Other current episode | F33.8 |
|  | Unspecified current depressive disorder | F33.9 |
|  | **Bipolar disorder diagnosis** | F30, F31 |
|  | **Anxiety disorder diagnosis** | F4 |
|  | **Schizophrenia diagnosis** | F2 |
|  | **Alcohol use disorder diagnosis** | F10 |
|  | **Substance abuse disorder (excl. alcohol) diagnosis** | F11-F19 |
|  | **ADHD diagnosis** | F90 |
|  | **Autism spectrum disorder diagnosis** | F84 |
|  | **Suicide history** | X60-X84; Y10-Y34 |
|  | **Antipsychotic medication** | N05A |
|  | **Hypnotics and sedatives medication** | N05B excl. N05BA, N05C |
|  | **Benzodiazepine medication** | N05BA |
|  | **Antiepileptic medication** | N03A |
|  | **ADHD medication** | N06B |
| **Time-varying confounders** | **Non-SSRI antidepressants** | N06A, excluding N06AB |
|  | **Benzodiazepines** | N05BA |
|  | **Any other psychotropic drug** | N02A, N03A, N05A, N05B excluding N05BA, N05C, N06B, N06D, N07B |

## **Table S4. Distribution of weights in analyses with 12 weeks’ follow-up, before and after truncation**

| **Weight^a^** | **Minimum** | **1^st^ quartile** | **Median** | **Mean** | **3^rd^ quartile** | **Maximum** |
| --- | --- | --- | --- | --- | --- | --- |
| SW^A^ – before truncation | 0.402 | 0.814 | 0.934 | 1.001 | 1.056 | 12.506 |
| SW^A^ – after truncation | 0.402 | 0.814 | 0.934 | 0.994 | 1.056 | 2.504 |
| SW^N^ – before truncation | 0.373 | 0.999 | 1.000 | 1.000 | 1.000 | 4.022 |
| SW^N^ – after truncation | 0.373 | 0.999 | 0.999 | 0.999 | 1.000 | 1.300 |
| SW^A^ * SW^N^ after truncation | 0.193 | 0.818 | 0.933 | 0.994 | 1.052 | 3.256 |
| ^a^ See Supplementary Methods for definitions of the weights | | | | | | |

## **Table S5. Per-protocol analyses over 12 weeks, stratified by sex and age**

|  | **N events, initiators** | **N events, non-initiators** | **Absolute risk, initiators % (95% CI)** | **Absolute risk,**  **non-initiators % (95% CI)** | **Risk difference % (95% CI)** | **Risk ratio (95% CI)** |
| --- | --- | --- | --- | --- | --- | --- |
| **Female** | | | | | | |
| **Overall** | 118 | 169 | 0.48 (0.31,0.65) | 0.25 (0.19,0.32) | 0.23 (0.05,0.41) | 1.91 (1.23,2.94) |
| **6-17 years** | 40 | 44 | 3.27 (1.05,5.50) | 0.80 (0.40,1.20) | 2.47 (0.19,4.76) | 4.09 (1.71,9.80) |
| **18-24 years** | 46 | 45 | 0.92 (0.40,1.43) | 0.46 (0.24,0.68) | 0.46 (-0.10,1.02) | 1.99 (0.94,4.22) |
| **25-39 years** | 25 | 45 | 0.20 (0.05,0.36) | 0.16 (0.09,0.24) | 0.04 (-0.13,0.21) | 1.25 (0.52,3.00) |
| **40-49 years** | 6 | 19 | 0.09 (-0.03,0.21) | 0.16 (0.04,0.28) | -0.07 (-0.24,0.10) | 0.56 (0.07,4.74) |
| **50-59 years** | 1 | 16 | 0.01 (-0.03,0.05) | 0.16 (0.03,0.29 | -0.15 (-0.28,-0.01) | 0.09 (0.00,48730.24) |
| **Male** | | | | | | |
| **Overall** | 70 | 136 | 0.45 (0.26,0.65) | 0.33 (0.24,0.42) | 0.12 (-0.09,0.33) | 1.38 (0.83,2.27) |
| **6-17 years** | 5 | 17 | 0.91 (-0.69,2.51) | 0.54 (0.12,0.96) | 0.37 (-1.27,2.01) | 1.68 (0.06,49.82) |
| **18-24 years** | 26 | 25 | 0.84 (0.28,1.39) | 0.37 (0.12,0.61 | 0.47 (-0.13,1.08) | 2.29 (0.88,5.93) |
| **25-39 years** | 23 | 48 | 0.39 (0.11,0.67 | 0.30 (0.16,0.44) | 0.10 (-0.23,0.42) | 1.32 (0.54,3.24) |
| **40-49 years** | 9 | 23 | 0.21 (-0.02,0.44) | 0.28 (0.09,0.47) | -0.07 (-0.37,0.23) | 0.75 (0.20,2.74) |
| **50-59 years** | 7 | 23 | 0.31 (-0.11,0.73 | 0.34 (0.08,0.59) | -0.02 (-0.53,0.48) | 0.93 (0.17,4.99) |

## **Table S6. Intention-to-treat and per-protocol analyses over 52 weeks**

|  | **N events, initiators** | **N events, controls** | **Absolute risk, initiators % (95% CI)** | **Absolute risk, non-initiators % (95% CI)** | **Risk difference % (95% CI)** | **Risk ratio (95% CI)** |
| --- | --- | --- | --- | --- | --- | --- |
| **Intention-to-treat ^a^** | 491 | 851 | 1.06 (0.99, 1.12) | 0.76 (0.73,0.79) | 0.30 (0.22,0.37) | 1.39 (1.16,1.66) |
| **Per-protocol ^b^** | 332 | 680 | 1.29 (1.00, 1.59) | 0.67 (0.58, 0.76) | 0.62 (0.32, 0.93) | 1.93 (1.49, 2.49) |
| ^a^ IPW weighted for baseline treatment assignment using baseline confounders  ^b^ IPW weights for baseline treatment assignment (using baseline confounders only) are multiplied by time-varying weights for weighting by treatment adherence (using baseline confounders and time-varying indicators of treatment with other medications) | | | | | | |

## **Table S7. Per-protocol analyses over 12 weeks, with alternative definition of events, alternative definition of treatment period length, or additional time-varying variables**

|  | **N events, initiators** | **N events, non-initiators** | **Risk among initiators % (95% CI)** | **Risk among non-initiators % (95% CI)** | **Risk difference % (95% CI)** | **Risk Ratio**  **(95% CI)** |
| --- | --- | --- | --- | --- | --- | --- |
| **Considering only suicidal behavior events of known intent** | 167 | 262 | 0.42 (0.30,0.54) | 0.24 (0.19,0.29) | 0.18 (0.05,0.30) | 1.74 (1.23,2.47) |
| **Treatment definition: assuming that individuals use one SSRI pill per day** | 160 | 305 | 0.41 (0.29,0.53) | 0.28 (0.23,0.34) | 0.12 (0.00,0.25) | 1.44 (1.02,2.04) |
| **With additional adjustment for time-varying diagnoses ^a^** | 188 | 305 | 0.47 (0.35,0.60) | 0.28 (0.23,0.34) | 0.19 (0.05,0.33) | 1.68 (1.20,2.36) |
| ^a^ The time-varying diagnoses additionally included: alcohol use disorder, anxiety disorder, bipolar disorder, psychotic disorder, substance use disorder (excluding alcohol). See Table S3 for diagnosis codes. | | | | | | |

## **Table S8. Per-protocol analyses over 12 weeks using alternative grace periods**

| **Grace period** | **N events**  **initiators** | **N events**  **non-initiators** | **Absolute risk**  **initiators % (95% CI)** | **Absolute risk non-initiators % (95% CI)** | **Risk difference % (95% CI)** | **Risk ratio (95% CI)** |
| --- | --- | --- | --- | --- | --- | --- |
| **7 days ^a^** | 132 | 319 | 0.44 (0.30,0.58) | 0.27 (0.22,0.32) | 0.17 (0.02,0.31) | 1.62 (1.13,2.32) |
| **14 days ^b^** | 155 | 318 | 0.44 (0.32,0.56) | 0.28 (0.23,0.33) | 0.16 (0.03,0.30) | 1.59 (1.14,2.21) |
| **28 days**  **(main analysis)** | 188 | 305 | 0.47 (0.34,0.60) | 0.28 (0.23,0.34) | 0.19 (0.05,0.33) | 1.69 (1.20,2.36) |
| **84 days ^c^** | 227 | 250 | 0.47 (0.35,0.59) | 0.25 (0.20,0.31) | 0.22 (0.09,0.35) | 1.86 (1.33,2.58) |
| ^a^ N initiators: 43,594; N non-initiators: 118,686  ^b^ N initiators: 47,859; N non-initiators: 114,421  ^c^ N initiators: 60,674; N non-initiators: 101,527 | | | | | | |

## **Table S9. Intention-to-treat and per-protocol analyses using the cloning-censoring-weighting approach**

|  | **N events, initiators** | **N events, non-initiators** | **Absolute risk, initiators % (95% CI)** | **Absolute risk, non-initiators % (95% CI)** | **Risk difference %**  **(95% CI)** | **Risk ratio (95% CI)** |
| --- | --- | --- | --- | --- | --- | --- |
| **Intention-to-treat** | 372 | 349 | 0.40 (0.35, 0.45) | 0.30 (0.26, 0.33) | 0.11 (0.04, 0.16) | 1.36 (1.15, 1.58) |
| **Per-protocol** | 354 | 349 | 0.53 (0.46, 0.61) | 0.36 (0.31, 0.43) | 0.17 (0.07, 0.26) | 1.47 (1.15, 1.78) |

## **Figure S1. Intention-to-treat risk curves over 52 weeks in initiators and non-initiators**


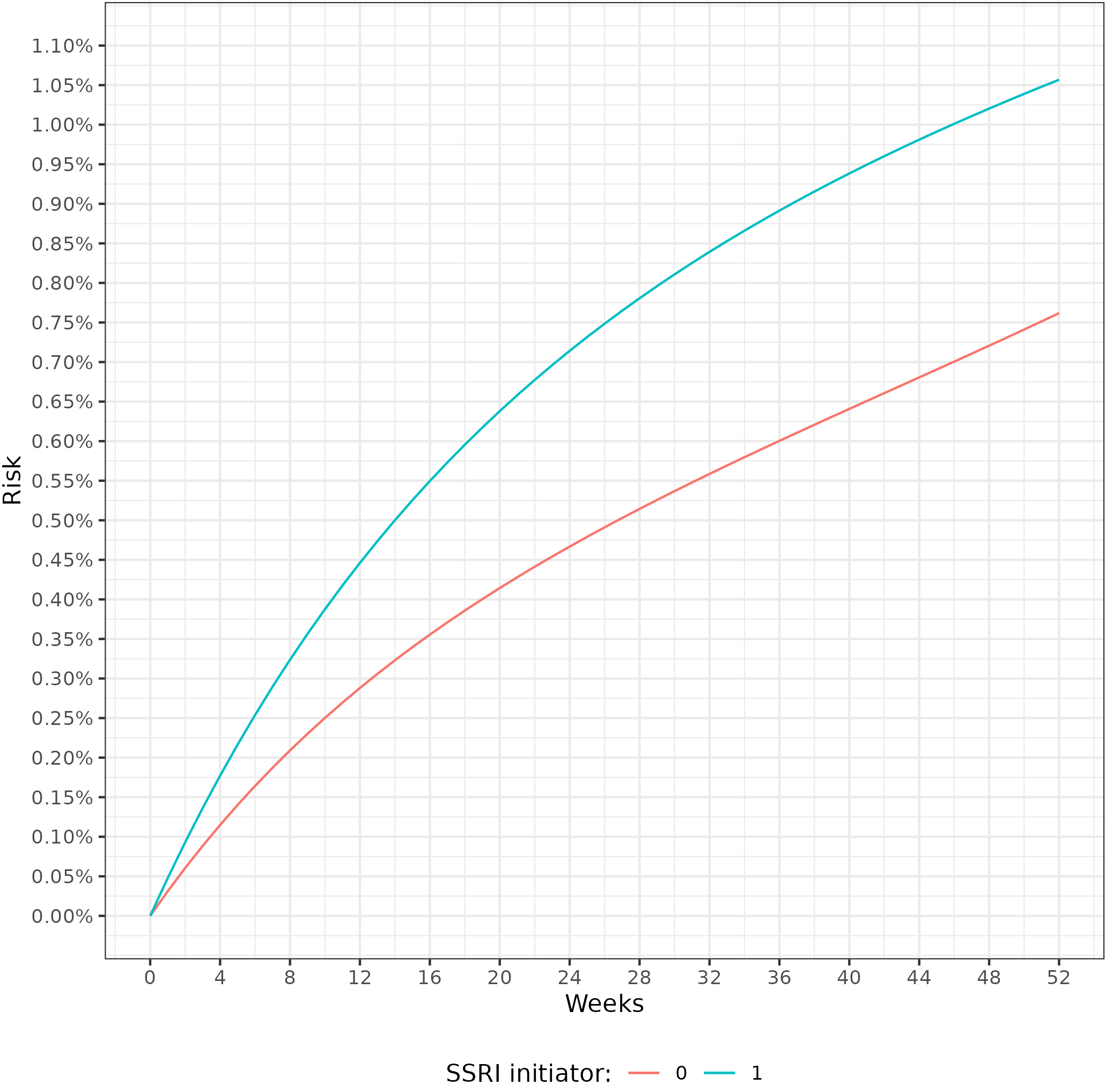


## **Supplementary methods**

**Main target trial emulation – SSRI initiation versus non-initiation**

We estimated intention-to-treat (ITT) and per-protocol (PP) effects for the main target trial emulation. The ITT analysis required adjustment for baseline characteristics; the PP analysis required adjustment for both baseline and time-varying characteristics. We did this by inverse probability weighting.

For both ITT and PP analyses, we adjusted for baseline confounding by applying stabilized inverse probability weights (SW^A^) for everyone included in the cohort. The numerator of the weight is the probability of receiving the observed treatment (*A*). The denominator of the weights is the probability that, given baseline confounders (*L*), an individual receives their observed treatment (*A*).^2^

𝑆W^A^ = Pr(𝐴) / Pr(𝐴|𝐿)

For the PP analysis, we additionally had to account for whether individuals remained adherent to their assigned treatment, by applying stabilized inverse probability weights (SW^N^), truncated at the 99^th^ percentile. The numerator of this weight is the probability of remaining adherent (*N*) to the assigned treatment given baseline confounders. The denominator of the weight is the probability of remaining adherent given baseline (*L*) and time-updated confounders (*M*).^3^

𝑆W^N^= Pr(*N*|*L*) / Pr(*N*|𝐿,*M*)

Both SW^A^ and SW^N^ weights are multiplied over time within individuals to get the weight at time *t* for a given individual (note that the SW^A^ weight remains the same within an individual over time). At this stage, the weights are truncated at the 99^th^ percentile, to avoid outliers having an outsize impact on estimates

Then, the pooled logistic regression model is fitted: in the ITT analysis, it is weighted by SW^A^; in the PP analysis, it is weighted by SW^A^*SW^N^.^3^

E([𝑌*_t_*_+1_ = 0|𝐴, 𝑌*_t_* = 0]) = α_0,_*_t_* + α_1_𝐴 + α_2_𝐴*t* + α_3_𝐴*t*^2^

Y_t+1_ is an indicator of whether the outcome occurred at time *t*+1; *A* is an indicator of the treatment group; α_0,t_ is the time-varying intercept estimated as a constant plus linear and quadratic terms at time *t*.

The survival at each time *t* of follow-up for each treatment strategy can subsequently be estimated by multiplying the predicted probabilities over time.

The risk at a given time *t* can then be estimated by subtracting the estimated survival from 1.

We used nonparametric bootstrapping with 500 samples to estimate 95% confidence intervals.

**References**

1. Zhou Z, Rahme E, Abrahamowicz M, et al. Survival bias associated with time-to-treatment initiation in drug effectiveness evaluation: a comparison of methods. *American journal of epidemiology* 2005;162(10):1016-23.

2. Matthews AA, Szummer K, Dahabreh IJ, et al. Comparing effect estimates in randomized trials and observational studies from the same population: an application to percutaneous coronary intervention. *Journal of the American Heart Association* 2021;10(11):e020357.

3. Hernán MA, Lanoy E, Costagliola D, et al. Comparison of dynamic treatment regimes via inverse probability weighting. *Basic & clinical pharmacology & toxicology* 2006;98(3):237-42.
